# Supplementary material for: Intra-strain genetic heterogeneity in Toxoplasma gondii ME49: Oxford Nanopore long-read sequencing reveals copy number variation in the ROP8-ROP2A locus
Source: BMC Genomics. 2025 Dec 3;26:1094. doi: 10.1186/s12864-025-12372-5 (PMC12690857; doi:10.1186/s12864-025-12372-5)
Supplement: Supplementary file 2 — Supplementary Material 2. [file 12864_2025_12372_MOESM2_ESM.pdf]

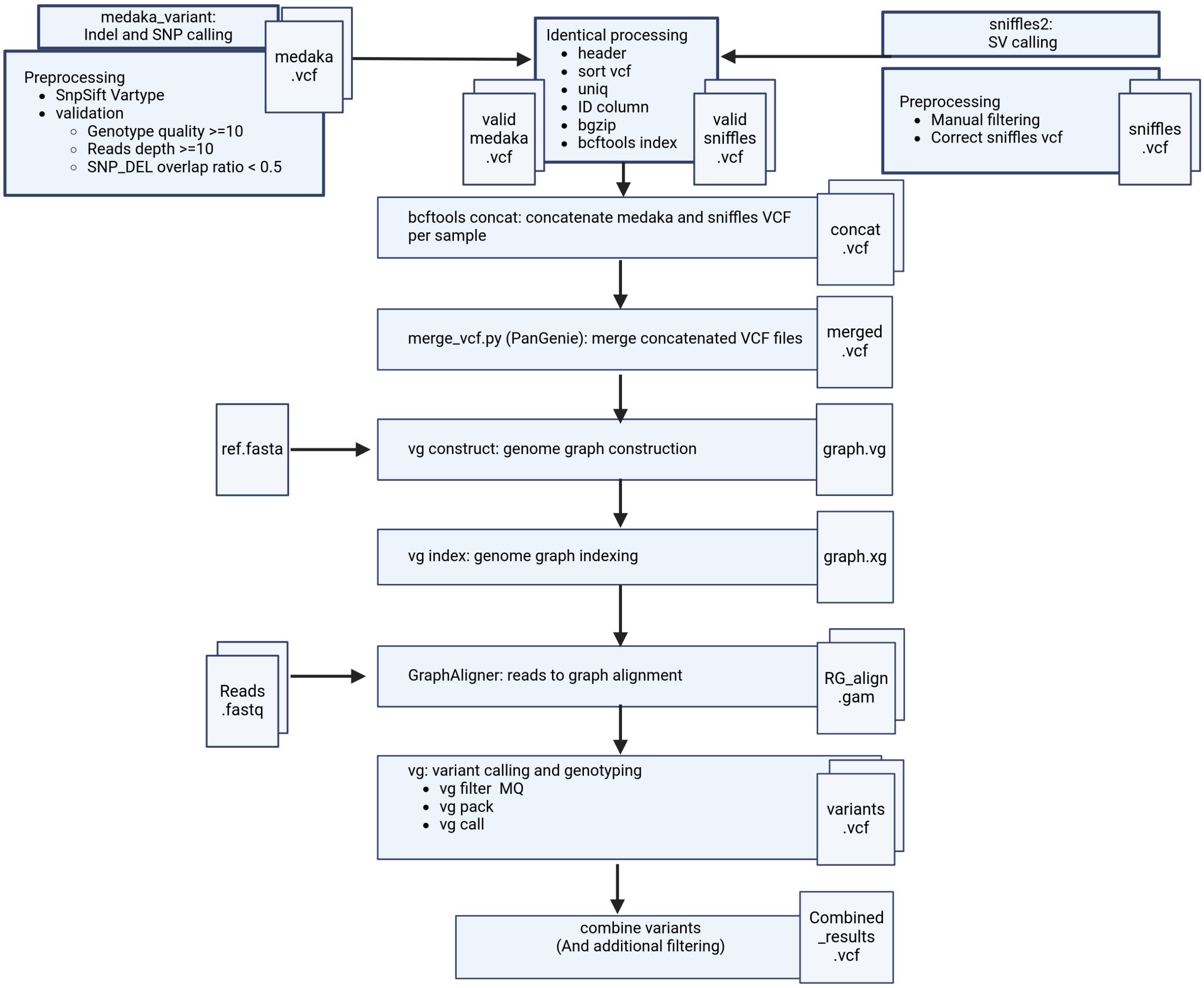

Supplementary Figure S1:
Flowchart of the Snakemake pipeline used in the variant analysis. Created in https://BioRender.com

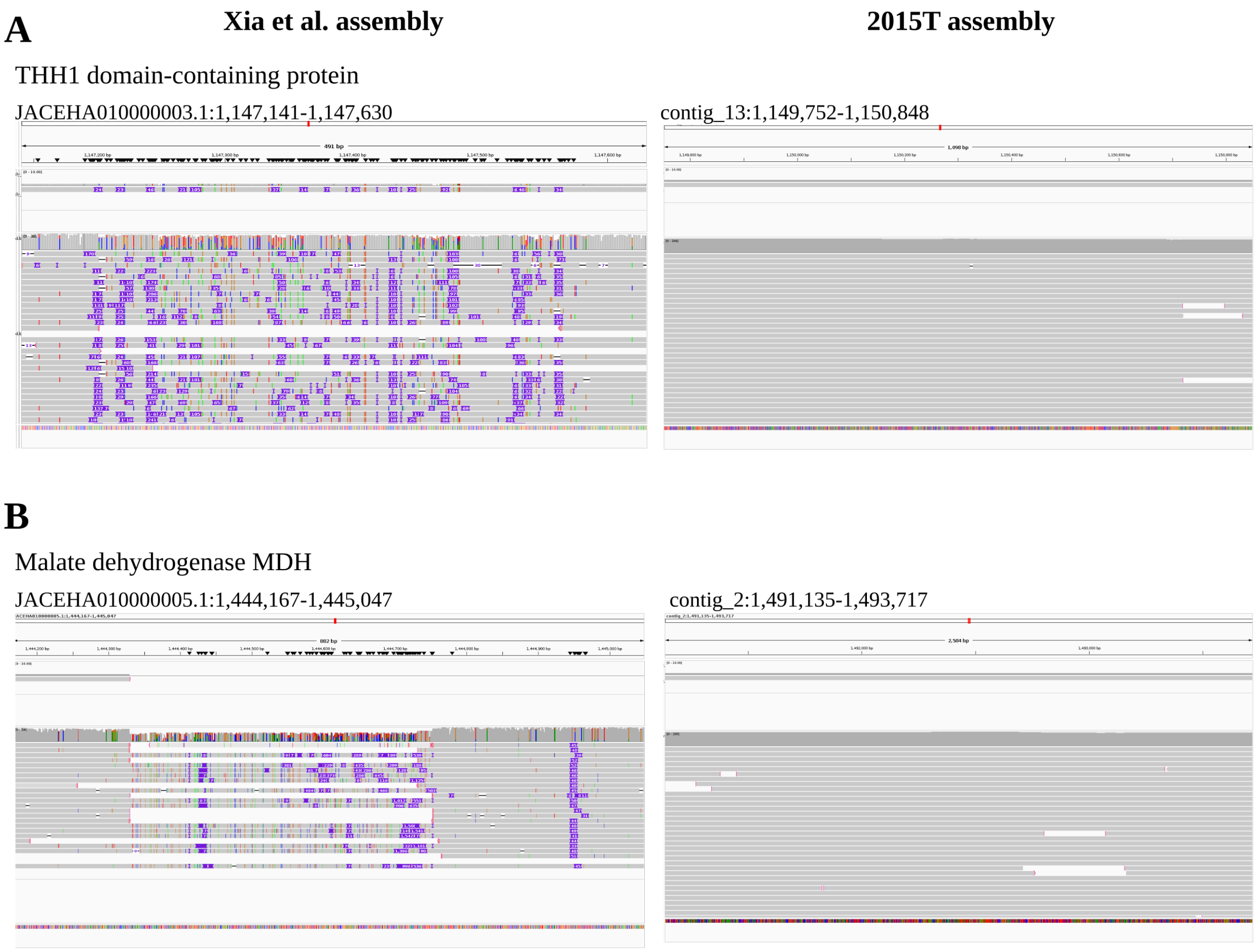

Supplementary Figure S2: Improved alignment of sequencing reads and ToxoDB reference gene sequence in the 2015T assembly compared to the Xia et al. assembly.

In each IGV panel, the top track shows the gene from ToxoDB, and the bottom track displays the long reads used to construct each assembly.

(A) Read alignment over the *THH1 domain-containing* gene *(TGME49_222310)*. The Xia et al. assembly exhibits a high density of mismatches and indels, whereas the 2015T assembly shows clean and consistent alignment. (B) Read alignment over the *Malate dehydrogenase (MDH)* gene *(TGME49_318430)*. In the Xia et al. assembly, extensive mismatches and gaps lead to incomplete mapping of the gene sequence. In contrast, the 2015T assembly enables smooth and concordant alignments, reflecting improved sequence accuracy.

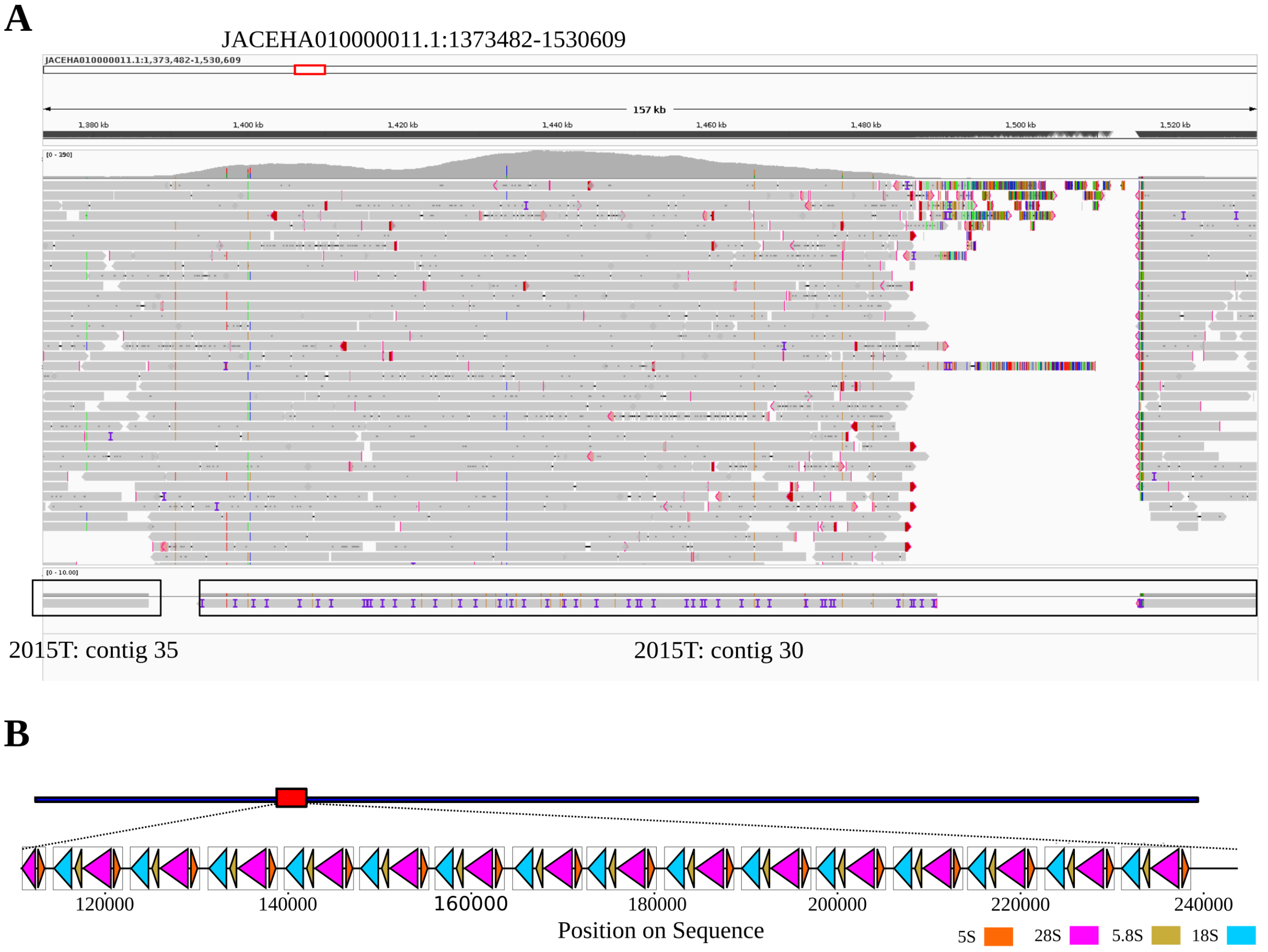

Supplementary Figure S3. Evidence of a likely misassembled rDNA repeat region in the Xia et al. assembly.
(A) IGV screenshot showing the alignment of reads used to construct the Xia et al. assembly (JACEHA010000011.1:137,482–1,530,609). A marked increase in read depth is observed in this region, accompanied by a lack of spanning reads, suggesting a potential misassembly. The bottom track shows the alignment of 2015T assembly contigs (contig_35 and contig_30). (B) Schematic representation of rDNA gene blocks annotated in the same region of the Xia et al. assembly.

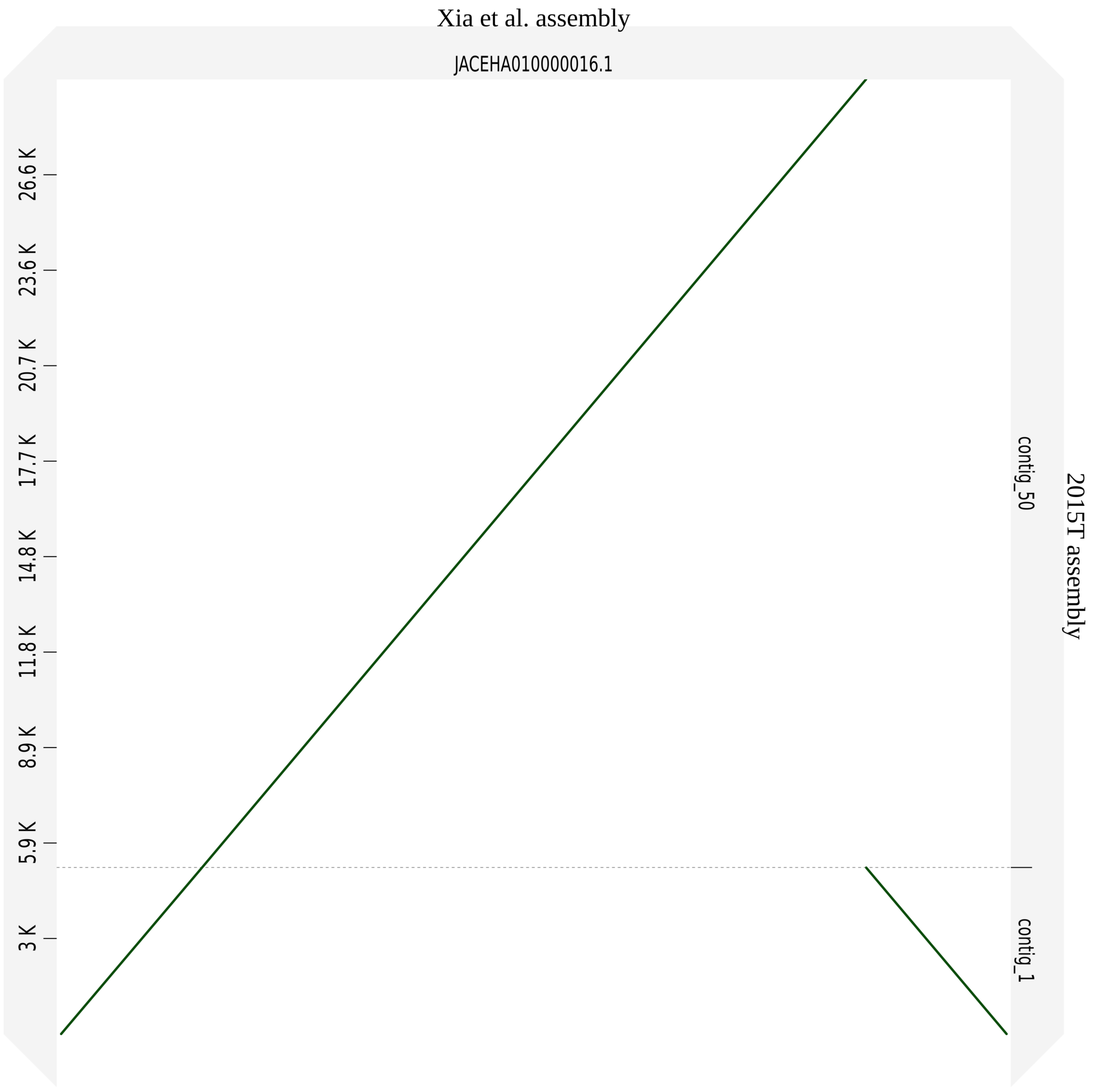

Supplementary Figure S4. Dot plot comparison of the apicoplast genome between the Xia et al. assembly and the 2015T assembly.
A dot plot generated using D-Genies shows alignment between the apicoplast contig from the Xia et al. assembly (JACEHA010000016.1) and the 2015T assembly. A single contig from the 2015T assembly (contig_1) aligns to the Xia et al. contig in two segments: once in the forward direction and once in the reverse complement. This pattern is consistent with the presence of an inverted repeat in apicoplast genome.

A

2000B reads against 2015T assembly before modification

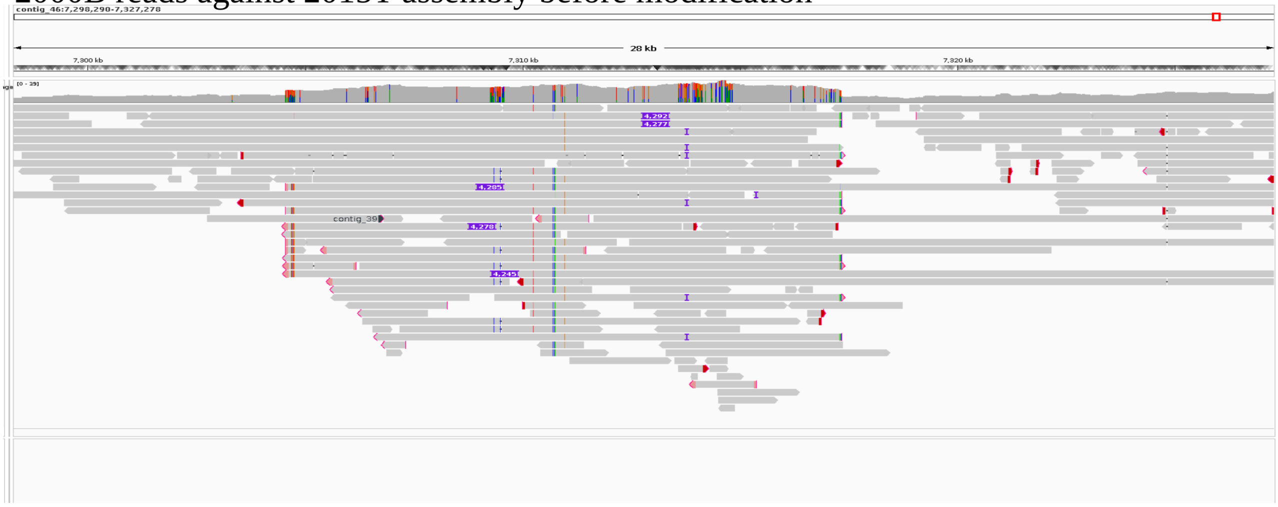

B

2000B reads against 2015T assembly after modification

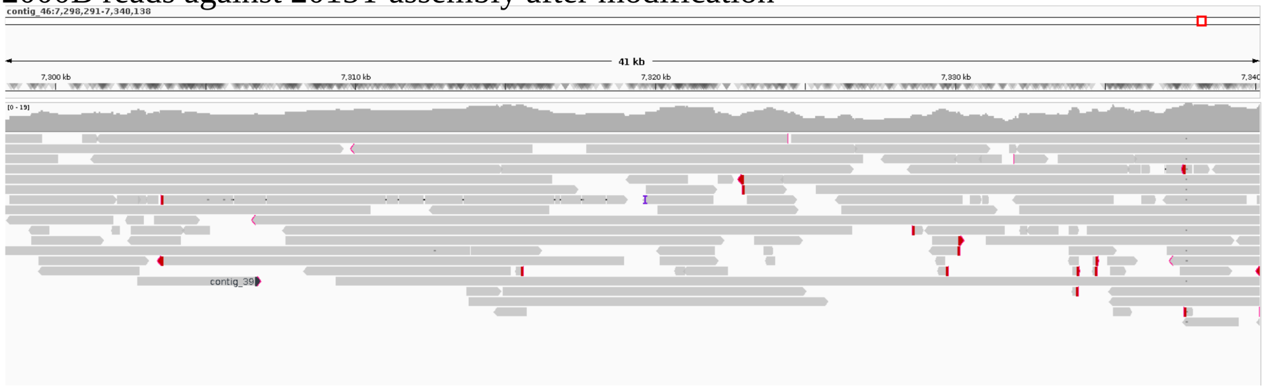

Supplementary Figure S5. Resolution of a structural variant in the 2000B isolate through reference modification.
(A) IGV snapshot showing 2000B reads aligned to the original 2015T assembly, displaying a region with abnormal coverage and extensive read clipping, consistent with a misrepresented structural variant. (B) Alignment of the same 2000B reads to the modified reference shows a uniform coverage profile with minimal clipping, indicating that the structural variant was correctly resolved. For clean visualization, secondary alignments and reads with mapping quality 0 (MAPQ0) were filtered out, and the coverage allele-fraction threshold was set to 0.5.

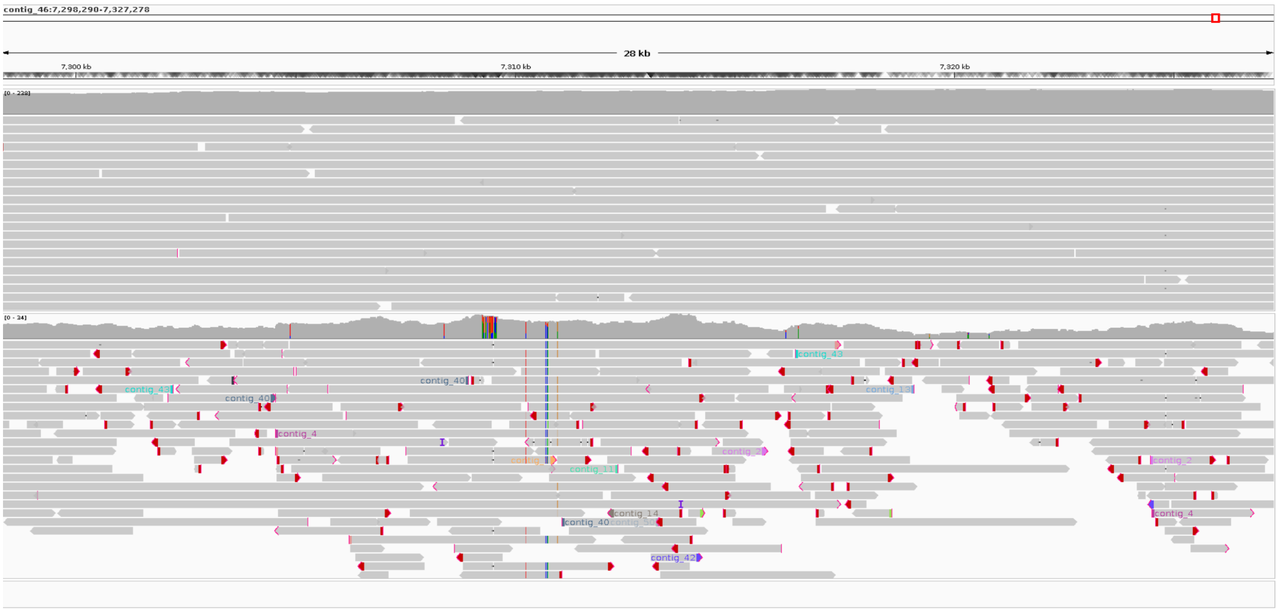

Supplementary Figure S6. Evidence for a structural variant in 2020T relative to the 2015T reference.
IGV screenshots showing read alignments of 2015T (top panel) and 2020T (bottom panel) to the 2015T assembly. While the 2015T reads align cleanly with uniform coverage, the 2020T reads show a mixed base pattern, where subsets of reads consistently support different genotypes across the same region. This pattern is similar to that observed in the 2000B alignment (Figure S5A) and is consistent with a collapsed repeat or unresolved structural variation in the reference assembly. For clean visualization, secondary alignments and reads with mapping quality 0 (MAPQ0) were filtered out, and the coverage allele-fraction threshold was set to 0.5.

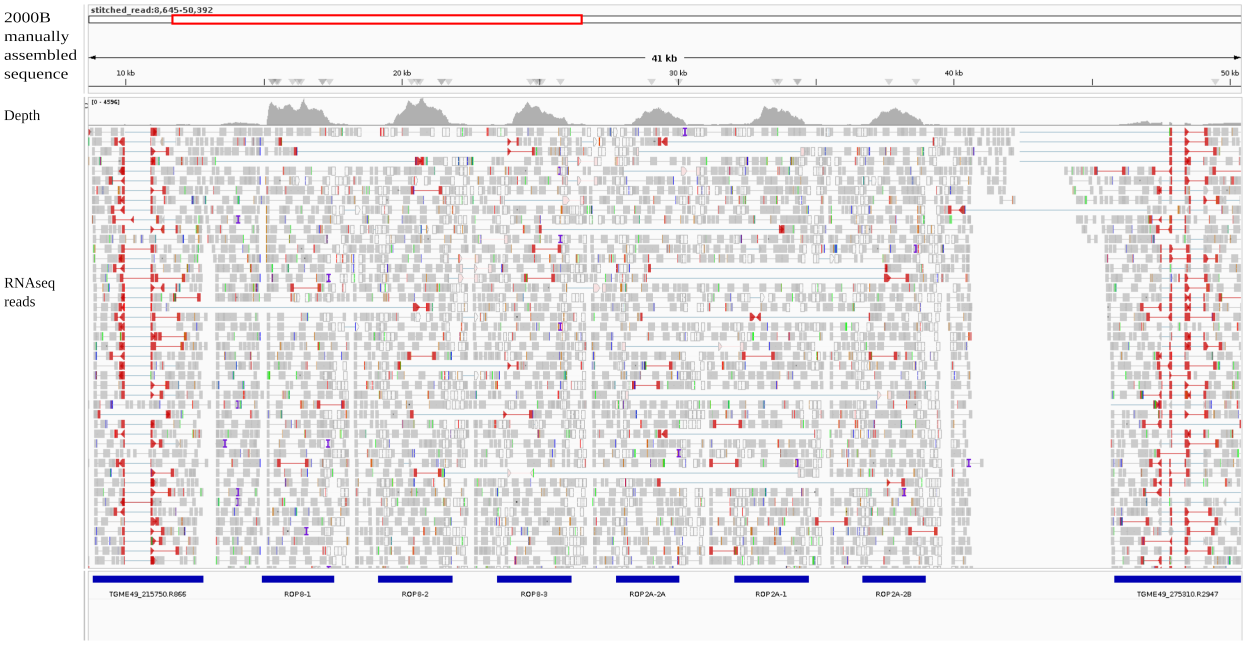

Supplementary Figure S7. RNA-seq read coverage across the *ROP8–ROP2A* locus in the 2000B.
IGV screenshot showing RNA-seq alignments from *T. gondii* ME49 mapped to the structurally resolved *ROP8–ROP2A* region of isolate 2000B. The coverage profile indicates detectable expression of ROP8-1, ROP8-2, ROP8-3, ROP2A-A, ROP2A-2B and ROP2A-1. The alignment is visualized with secondary alignments filtered out.

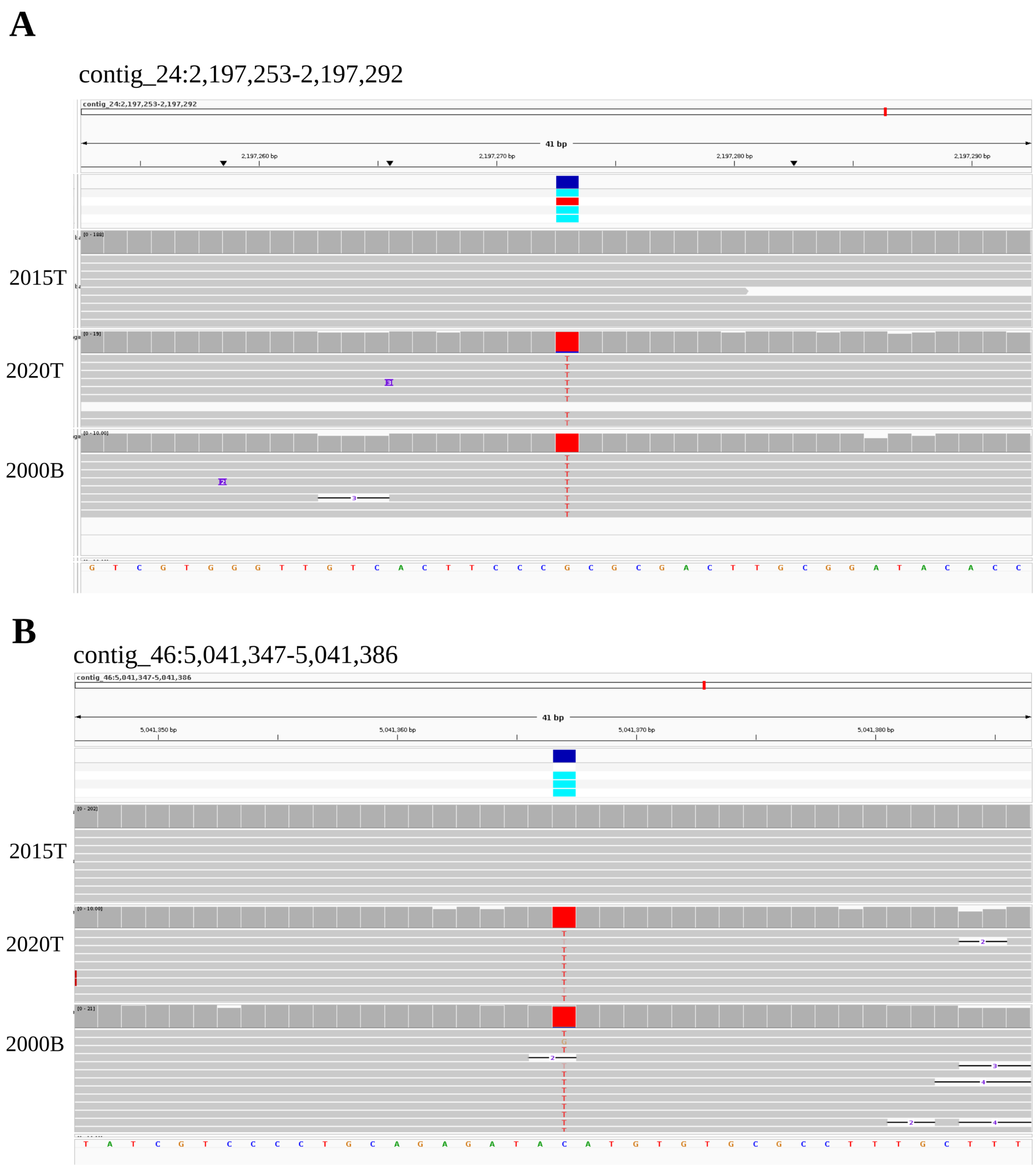

Supplementary Figure S8. Examples of genotype correction through graph-based variant calling.

(A) An example of a variant that was initially called only in isolate 2020T but missed in 2000B, despite clear evidence of its presence in both isolates. The graph-based genotyping approach correctly recovered the variant in both samples. (B) A case where a true variant in 2020T was erroneously filtered out during the Medaka-based filtering step. The variant was subsequently rescued by the graph-based genotyper, restoring the correct genotype.

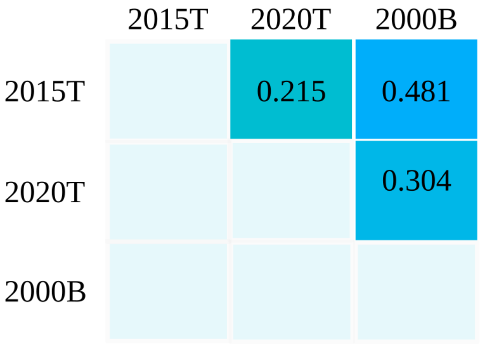

Supplementary Figure S9. Pairwise genetic similarity between isolates based on SNVs. Matrix showing inter-isolate genetic similarity calculated from shared single-nucleotide variants (SNVs). The highest level of similarity was observed between isolates 2000B and 2015T, indicating a closer genetic relationship relative to the other isolate pairs.

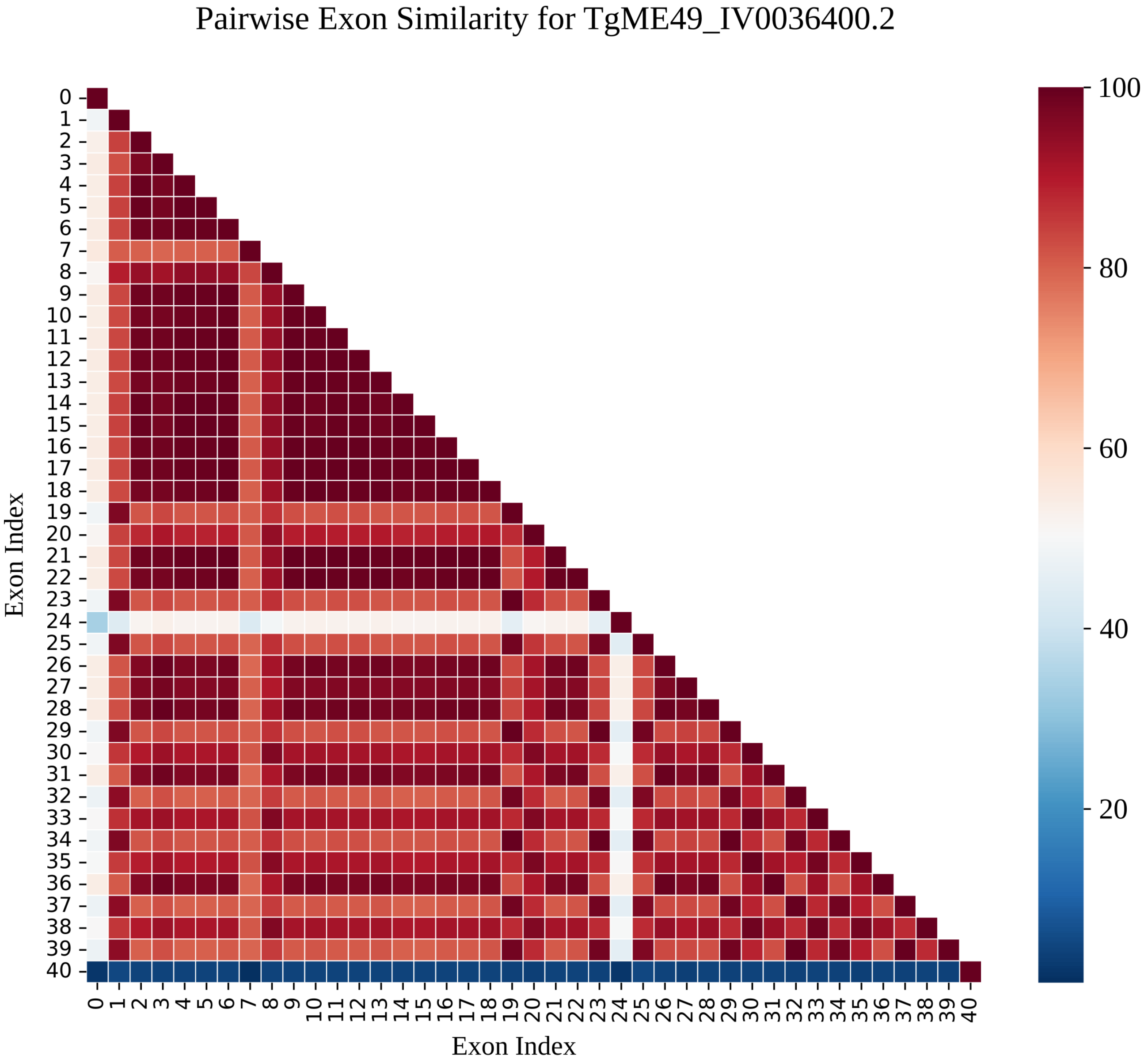

Supplementary Figure S10. Pairwise exon similarity matrix for *TgME49_IV0036400.2*

Heatmap showing the percent identity between all exon pairs of the gene *TgME49_IV0036400.2*. Exons were aligned in all-vs-all fashion, and similarity was calculated as percent identity over alignment length. The exon indices on both axes start at 0 and follow the exon order in the GTF annotation.
